# Supplementary figures and images for: Genome-wide identification of novel genes involved in Corynebacteriales cell envelope biogenesis using Corynebacterium glutamicum as a model
Source: PLoS One. 2020 Dec 31;15(12):e0240497. doi: 10.1371/journal.pone.0240497 (PMC7775120; doi:10.1371/journal.pone.0240497)

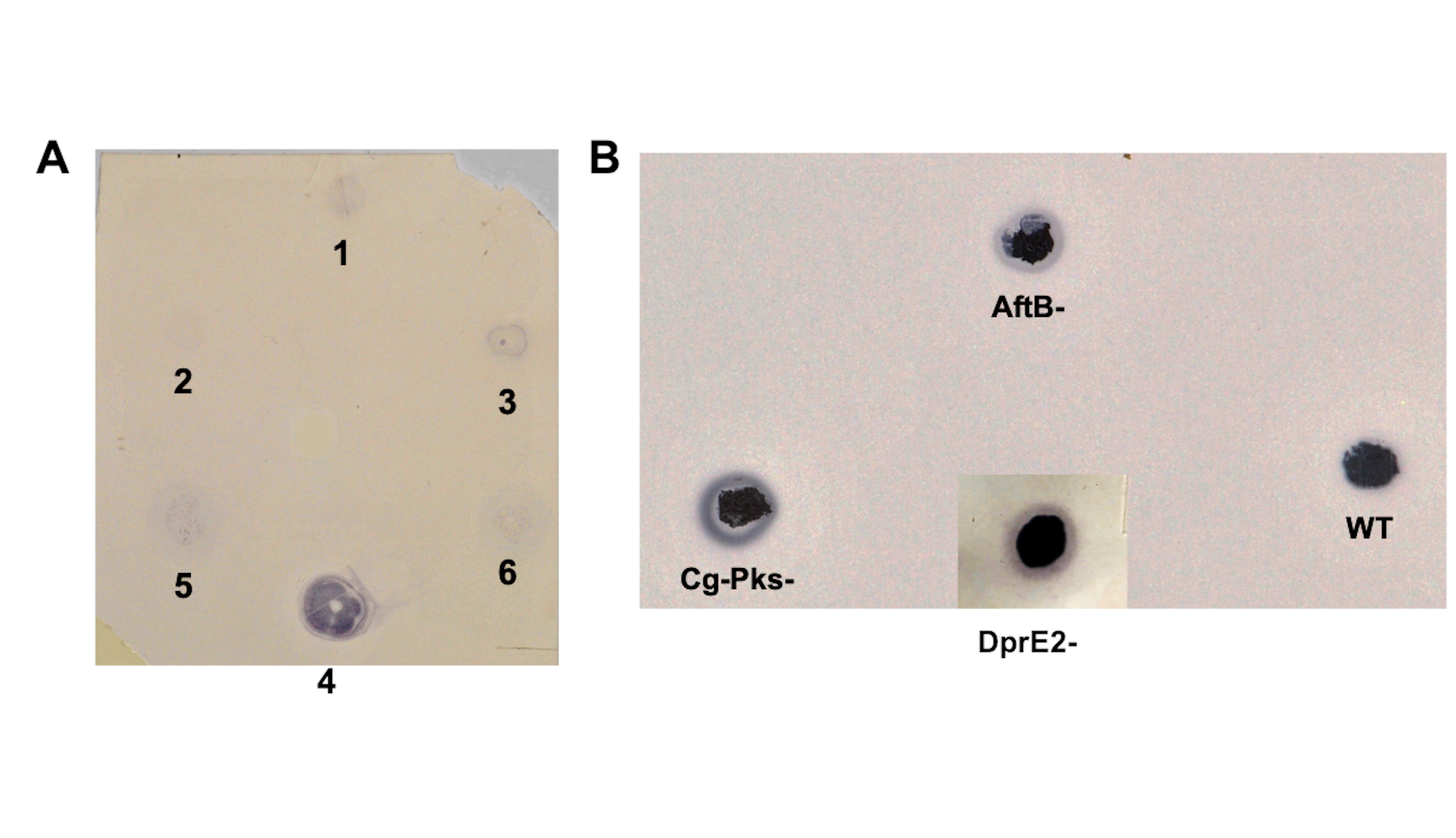

Supplement: S1 Fig — Identical experiments to that described in Fig 1 were repeated. (A) As a negative control, the pre-immune serum was used instead of the anti-CW serum, at the same dilution and incubation time. The strains used in this control (numbered 1 to 6 in the blot) are the same as those given in the table of Fig 1. (B) As positive controls, AftB- and DprE2- mutant strains were spotted and cultivated along with the Res167 WT strain and the Cg-Pks- mutant as controls. (TIF) [file pone.0240497.s001.tif]

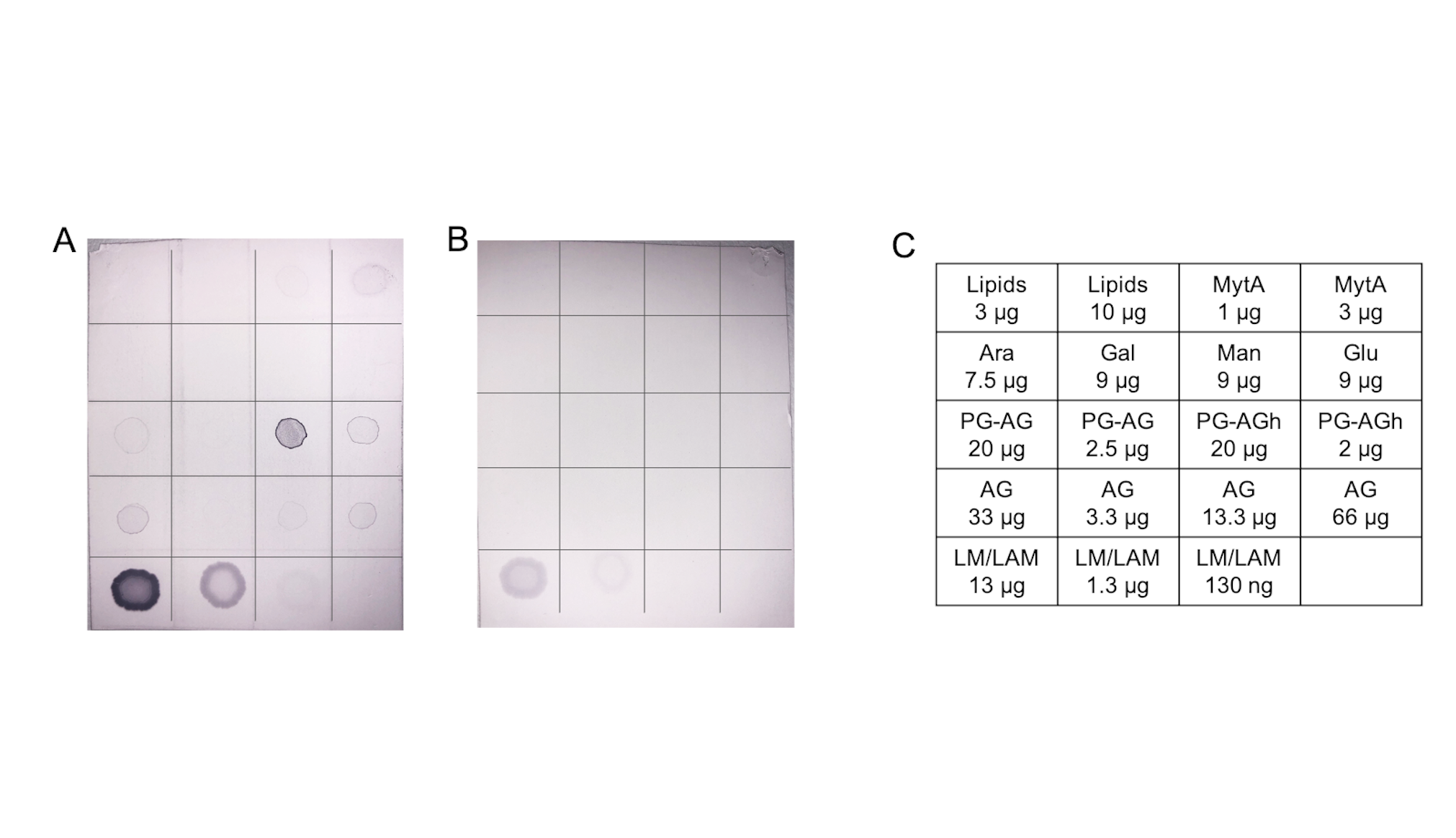

Supplement: S2 Fig — Different preparations containing envelope components or monosaccharide solutions, were spotted on a nitrocellulose membrane layered on a BHI plate. After 10 minutes incubation at room temperature, the membrane was recovered, treated with anti-CW antiserum and revealed as described in Materials and Methods (A). To obtain a replicate of the agar plate, immediately after removal of the nitrocellulose membrane, a new membrane was placed on the plate and treated as described in Materials and Methods. For more readability, the "imprint" sheet has been flipped to be read in the same direction as the sheet on which the components were dropped (B). Locations of the different samples and their respective quantities loaded on the membrane are indicated in (C). Lipids: extractible lipids from the ATCC13032 strain (see Fig 4B); MytA: purified MytAhis; AG, PG-AG and PG-AG hyrolyzed with H2SO4 (PG-AGh) were all prepared from the Cg-Pks- strain; Ara: arabinose; Gal: galactose; Man: mannose; Glu: glucose. (TIF) [file pone.0240497.s002.tif]

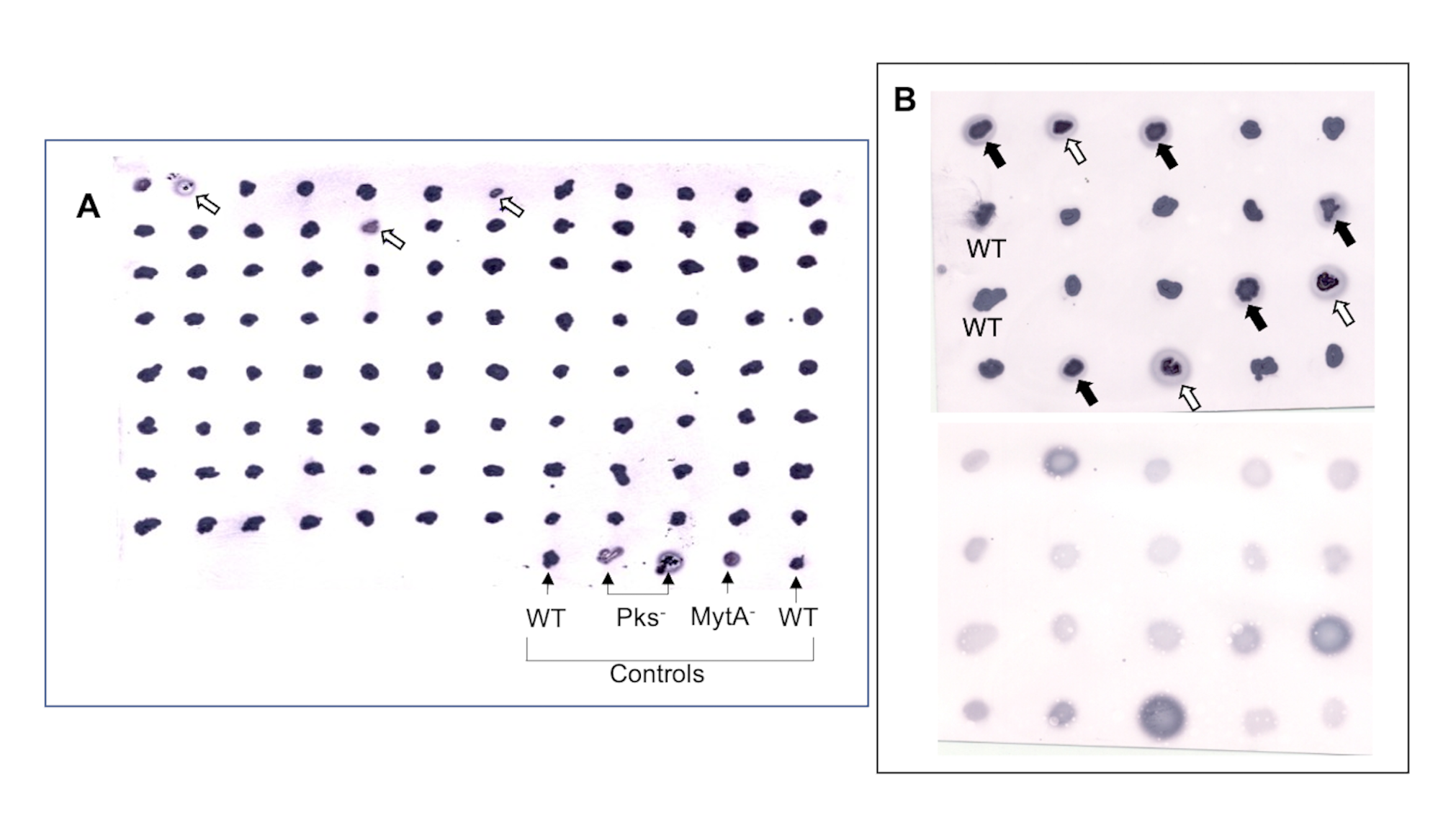

Supplement: S3 Fig — (A): Example of a membrane obtained after a first round of immunological screening. Nighty-six colonies corresponding to 96 different mutant strains, were grown on a nitrocellulose membrane layered on a BHI-plate. The membrane was treated with anti-CW antiserum and revealed as described in Materials and Methods. The lower line corresponds to control strains: 2 colonies of the WT strain 2262 and 3 colonies representing positive controls (Cg-Pks- and MytA-, strains inactivated in cg-pks and mytA genes respectively). The white arrows indicate 3 colonies selected for a second round of screening. (B): Example of a second-round immunological screening with mutants selected from the first round of immunological screening. On the top: the nitrocellulose membrane on which colonies have grown, on the bottom: the nitrocellulose membrane corresponding to the imprint of the agar plate. For more readability, the "imprint" sheet has been flipped to be read in the same direction as the sheet on which mutants grew. White and black arrows indicate mutants to which a score of 2 or 1 have been assigned, respectively. (TIF) [file pone.0240497.s003.tif]

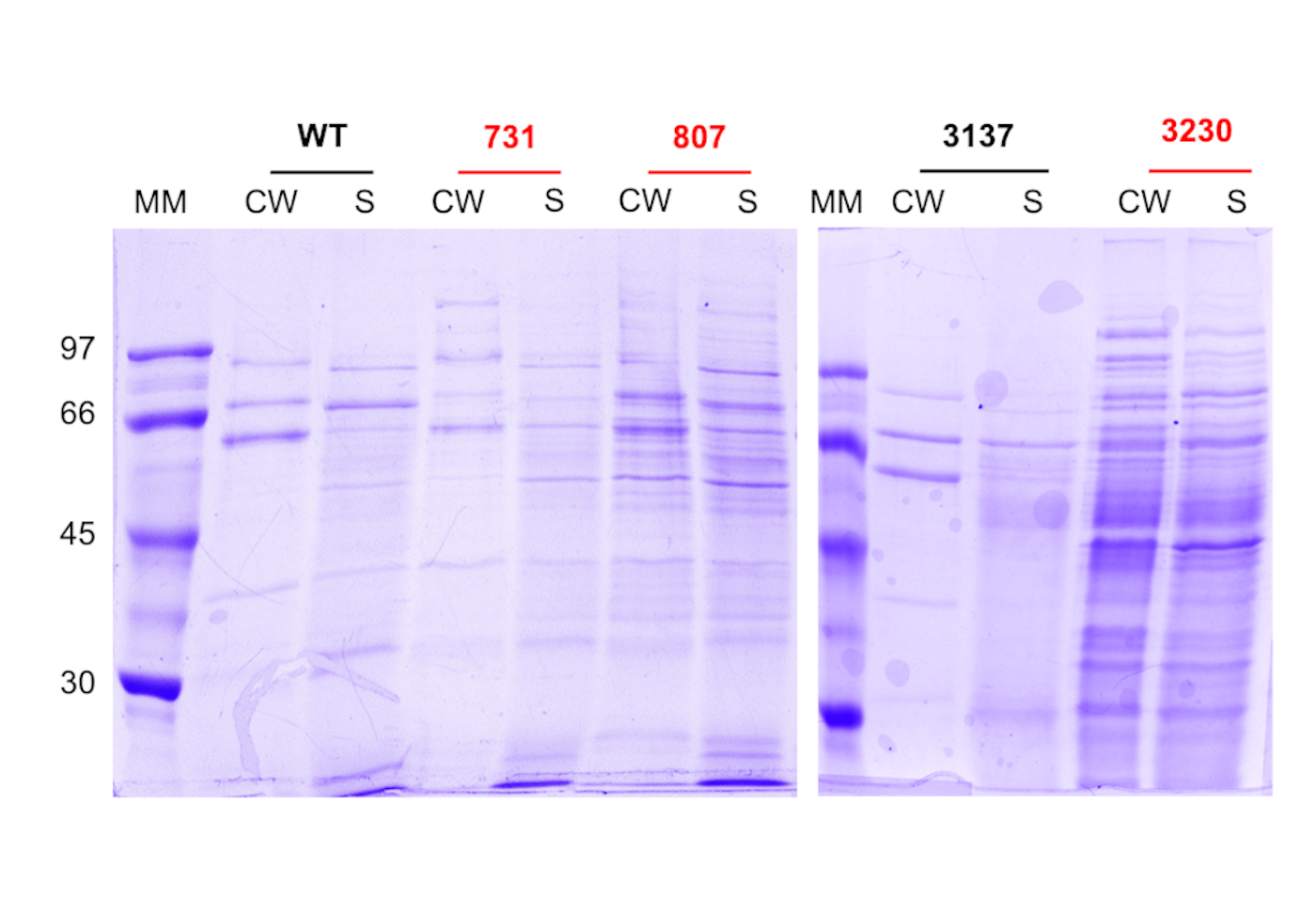

Supplement: S4 Fig — Procedures were performed as described in Materials and Methods. In this example, the mutants whose numbers are written in red (strains n° 731, 807 and 3230) have been scored 1 because of a visible alteration of their cell wall and extracellular protein profiles. The mutant 3137 was scored 0 because of the similarity of its protein profiles with those of the WT strain. S, supernatant containing the secreted proteins; CW, cell-wall fraction; MM, molecular mass markers (in kDa). (TIF) [file pone.0240497.s004.tif]
