# Supplementary material for: Genome-wide identification of novel genes involved in Corynebacteriales cell envelope biogenesis using Corynebacterium glutamicum as a model
Source: PLoS One. 2020 Dec 31;15(12):e0240497. doi: 10.1371/journal.pone.0240497 (PMC7775120; doi:10.1371/journal.pone.0240497)
Supplement: S1 Table — (DOCX) [file pone.0240497.s005.docx]

**S1 Table: Primers used in this study**

| **Primer name** | **Primer sequence** |
| --- | --- |
| *Inverse PCR primers* | |
| Isb01 | GTTATCCTAGGGGTGAGGTATAGTCGAGTGCGTC |
| Isb04 | TTATCCTAGGGGCAGTTGGTCCATAAGATCAATGC |
| CdsVIII | TATTTCGAAGGGGATCAAGCCTGATTG |
| CdsX | TATTTCGAATGGTTCGCTGGGTTTATC |
| M13 Rev | CAGGAAACAGCTATGAC |
| M13 F-20 | GTAAAACGACGGCCAG |
| *AP-PCR primers* | |
| ARB-4020 | GGCCACGCGTCGACTAGTACNNNNNNNNNNCGCCG |
| ARBq | GGCCACGCGTCGACTAGTAC |
| Isb012 | ATGGTGAGTCCTAGTTCG |
| Isb013 | ATGGGTAGAGCCTTTTGTTG |
| *Gene deletion primers* | |
| 1246-del1 | TTGAAGATCTCCTGGGATTCTTG |
| 1246-del2 | ATACCCGCGGCTACTTCCTAAC |
| 1246-del3 | ATCCCCGCGGTGTTTTCTTACCTC |
| 1246-del4 | TATGCTCGAGTAAACCAACCGTG |
| *Gene complementation primers* | |
| 1246-XhoI | TCCGCTCGAGGAACAAATTAAAGC |
| 1246-RcaI | CTGGTCATGACTGATCTTCATCCC |
| P1246-BglII | TTTGGAGATCTGTACTCGCATTATC |
| P1246-RcaI | CTGGTCATGAGAGTTTTACTTACCTTTCAAC |
