# Supplementary material for: Genome-wide identification of novel genes involved in Corynebacteriales cell envelope biogenesis using Corynebacterium glutamicum as a model
Source: PLoS One. 2020 Dec 31;15(12):e0240497. doi: 10.1371/journal.pone.0240497 (PMC7775120; doi:10.1371/journal.pone.0240497)
Supplement: S1 Raw images — (PDF) [file pone.0240497.s008.pdf]

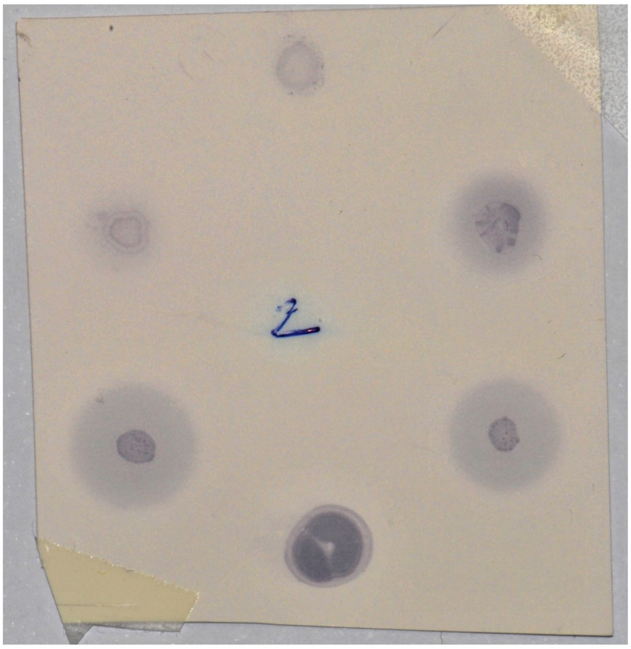

Immunoblot shown in Fig 1 (Photography)

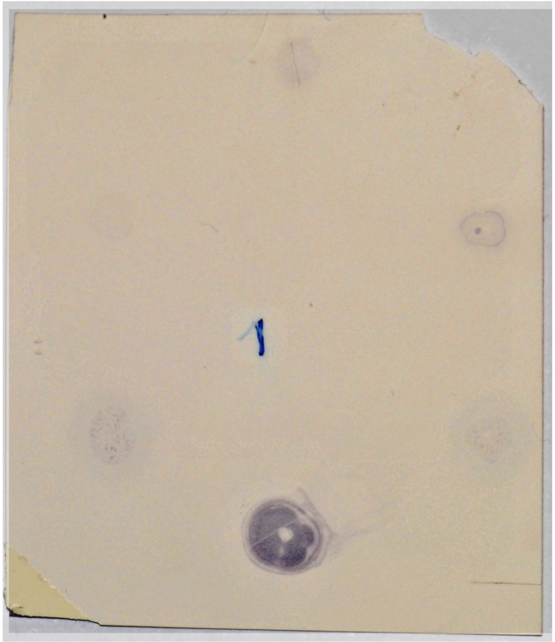

Immunoblot shown in S1-A Fig

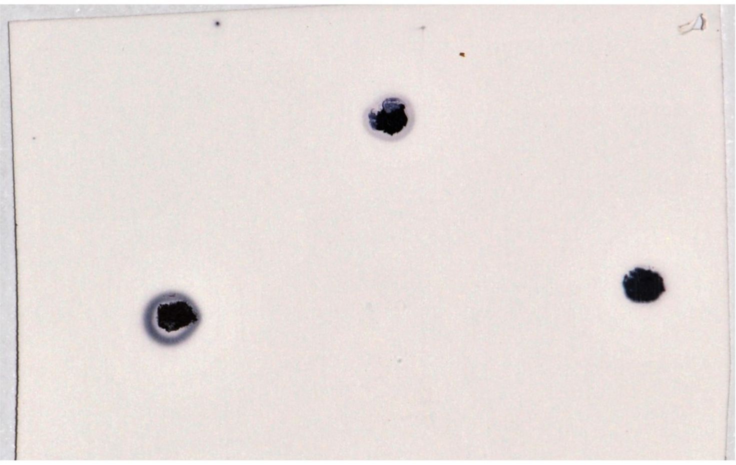

Immunoblot shown in S1-B Fig (AftB-, Cg-Pks- and WT strains), (Photography)

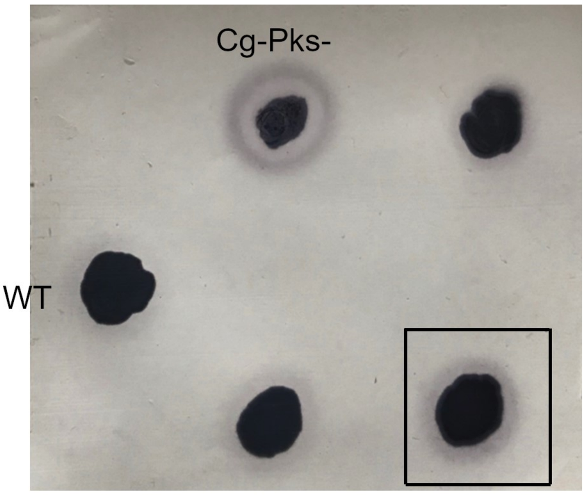

Part of the immunoblot used in S1-B Fig (surrounded in black) to show the DprE2- mutant strain. Two controls are shown in the same immunoblot (WT and Cg-Pks- strains). (Photography)

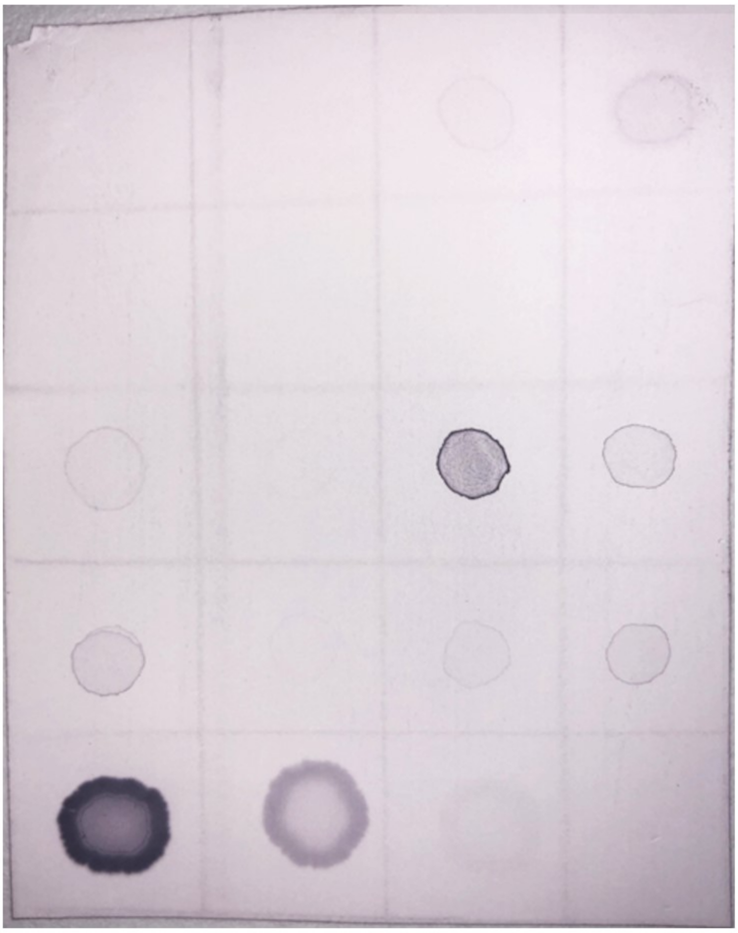

Immunoblot shown in S2-A Fig (photography)

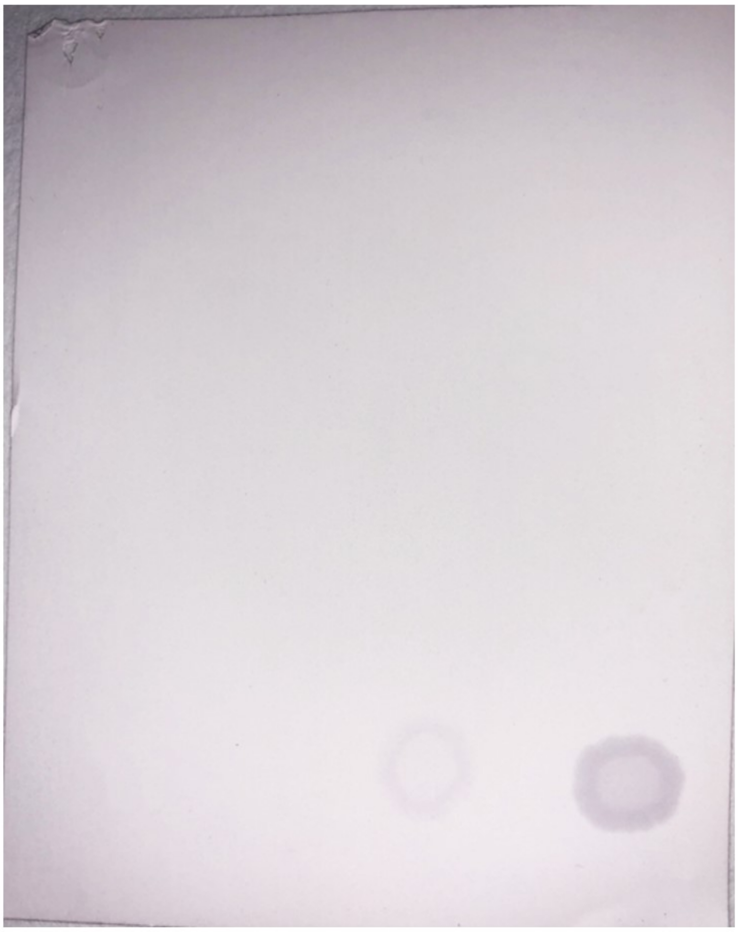

Immunoblot shown in S2-B Fig (photography)

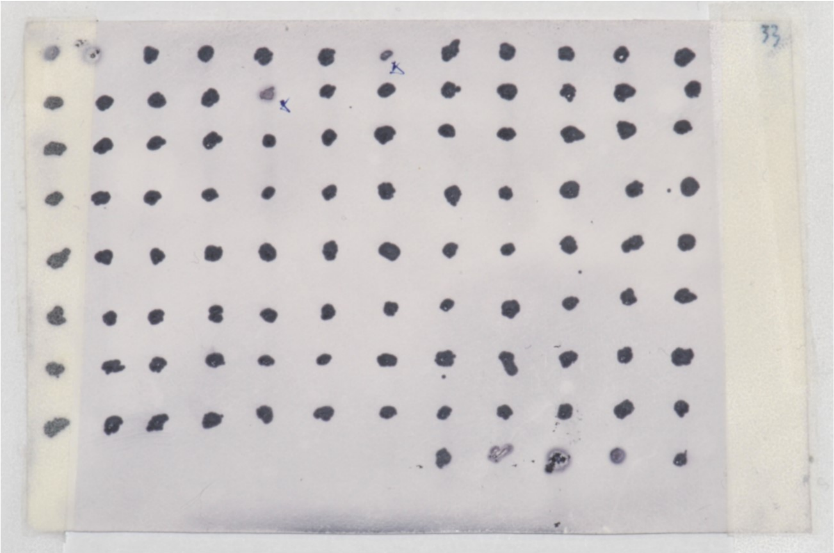

Immunoblot shown in S3-A Fig. (Photography)

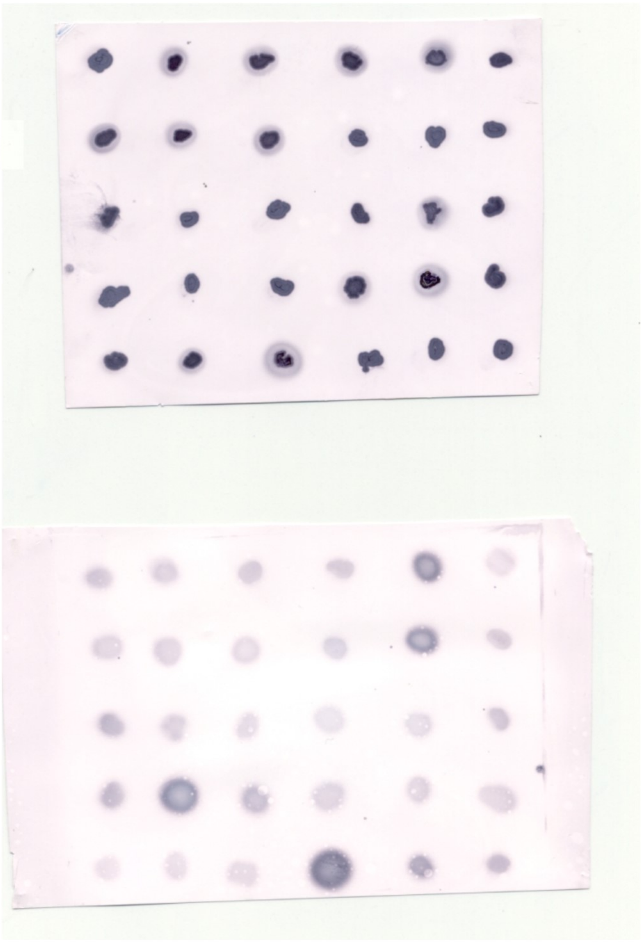

Immunoblots shown in S3-B Fig (photography)

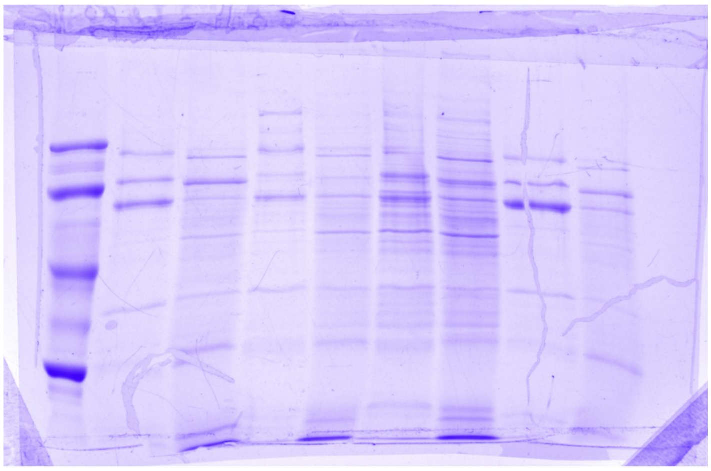

SDS PAGE shown in S4 Fig (left) (Image obtained with an Imager (Fusion FX, Vilber))

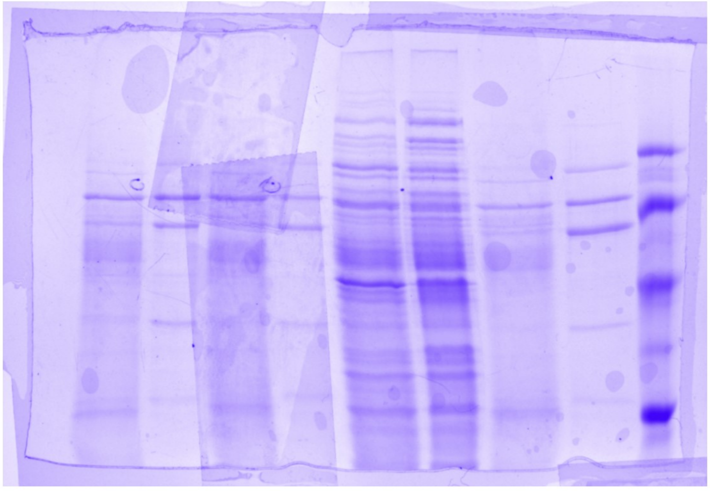

SDS PAGE shown in S4 Fig (right) (Image obtained with an Imager (Fusion FX, Vilber))
